# Mobile App Strategy to Facilitate Human Papillomavirus Vaccination Among Young Men Who Have Sex With Men: Pilot Intervention Study

**DOI:** 10.2196/22878

**Published:** 2020-11-04

**Authors:** Holly B Fontenot, Bradley Patrick White, Joshua G Rosenberger, Hailee Lacasse, Chokdee Rutirasiri, Kenneth H Mayer, Gregory Zimet

**Affiliations:** 1 School of Nursing and Dental Hygiene University of Hawaii at Manoa Honolulu, HI United States; 2 The Fenway Institute Boston, MA United States; 3 Connell School of Nursing Boston College Chestnut Hill, MA United States; 4 College of Health and Human Development Penn State University University Park, PA United States; 5 Proper Villains Boston, MA United States; 6 School of Arts and Sciences Boston College Chestnut Hill, MA United States; 7 Department of Medicine Beth Israel Deaconess Medical Center Boston, MA United States; 8 School of Medicine Harvard University Boston, MA United States; 9 Division of Adolescent Medicine School of Medicine Indiana University Indianapolis, IN United States

**Keywords:** human papillomavirus, men who have sex with men, vaccination, mobile health tool, mHealth

## Abstract

**Background:**

Mobile app-based interventions have been identified as potential facilitators for vaccination among young men who have sex with men (MSM).

**Objective:**

This pilot study aimed to test the feasibility of a theoretically informed mobile health (mHealth) tool designed to reduce health disparities and facilitate human papillomavirus (HPV) vaccination among a sample of young MSM.

**Methods:**

The development of the mHealth tool was guided by previous research, implementation intention theory, and design thinking. We recruited MSM aged 18-26 years through a popular online dating app and linked participants to our mHealth tool, which provided HPV vaccine information and fostered access to care.

**Results:**

A total of 42 young MSM participated in this pilot study in Boston, Massachusetts. Participants reported variable HPV knowledge (ie, high knowledge of HPV risk factors and low knowledge of HPV-related cancer risks for men) and positive vaccine beliefs and attitudes. Of those who were either unvaccinated, not up to date, or did not report vaccine status, 23% (8/35) utilized the mHealth tool to obtain HPV vaccination. Participants primarily utilized the tool’s (1) educational components and (2) capabilities facilitating concrete vaccine action plans.

**Conclusions:**

We recruited an underserved at-risk population of youth via an online dating app for our mHealth intervention that resulted in in-person health care delivery. This study was limited by enrollment challenges, including low willingness to download the mHealth tool to mobile devices.

## Introduction

In the United States, human papillomavirus (HPV) infection is a significant public health issue, and a disparity exists for men who have sex with men (MSM). As compared to men, in general, MSM experience a higher prevalence of HPV and HPV-related diseases [1]. HPV-related diseases among men include anogenital warts, anal cancer, penile cancer, oropharyngeal warts, and oropharyngeal cancer [2,3]. US national rates of HPV-related cancers are increasing among men [4,5], and rates of HPV-related anal cancers are the highest among young MSM [2,5,6].

Despite the availability of HPV vaccination for men since 2009, rates of HPV vaccination initiation and series completion among young MSM remain unacceptably low in the United States. In 2018, when this study was conducted in the United States, national vaccination rates (≥ 1 dose) in 13- to 17-year-old adolescent boys were low (41.7%) [7], and the rates were notably lower among young MSM (up to age 26 years; reported rates ranged from 4.9% to 13%) [8-10]. These rates contrasted sharply with the national rates of HPV vaccination in women at the time (60% adolescent girls aged 13-17 years had received ≥1 HPV vaccine dose) [7].

There are several factors that contribute to HPV vaccine disparities for men and young MSM, in particular. It is likely that the initial marketing of the cervical cancer vaccine for women combined with weak recommendations (or no recommendations) from health care providers for HPV vaccination for men led to these disparities among all men [11]. The US national HPV guidelines (from 2009 to 2019) recommended vaccination in a 3-dose series for all men up to age 21 years and up to age 26 years for young MSM. However, this sexual orientation–based vaccine recommendation was problematic, as (1) many young MSM (due to stigma and discrimination) do not disclose their sexual orientation to their health care providers [12,13] and (2) health providers do not routinely ask about sexual orientation [14]. This lack of open communication (assessment and disclosure) decreases the likelihood that appropriate health services are offered [12,15]. Lastly, young MSM also report low knowledge on HPV and the HPV vaccine, a lack of awareness of vaccine indications for men, and low perceived risk of HPV acquisition [16-18].

Overall, research on barriers to vaccination indicates that both attitudinal (eg, health beliefs) and logistical (eg, health care provider recommendation and concerns about cost) factors interfere with vaccine uptake [8,19,20]. Previous research indicates that young MSM report positive vaccine attitudes along with a belief that mobile app-based interventions can facilitate vaccination and access to care [16,21,22].

Young MSM tend to heavily utilize social media and mobile apps to seek sexual health information as well as sexual partners [23-25]. There is a rapid growth of mobile apps designed to facilitate meeting social/sexual partners, with recent estimates indicating at least 6.2 million MSM users nationally [23]. These extremely popular mobile social networks represent “virtual” communities of young MSM. Mobile social network/dating apps have been used to successfully recruit MSM for rectal microbicide [22] and sexual risk behavior research [23]. In addition, young MSM have overwhelmingly identified a willingness to participate in research if advertised on MSM mobile apps [18,21]. Therefore, recruitment of young MSM via a mobile social network for an app-based intervention [24] is an important strategy to consider for HPV vaccine promotion.

This pilot study evaluated a novel app-focused approach to facilitate HPV vaccination among young MSM. We connected with young MSM through popular dating apps and linked them to our mobile health (mHealth) tool, a web-enabled app designed to reduce MSM-specific barriers to vaccination (eg, knowledge/awareness, stigma, and discrimination) and overcome logistical barriers to HPV vaccination (eg, access to MSM-affirming health care and cost concerns).

## Methods

### mHealth Tool and Theory

The mHealth tool is a universally compatible web-enabled mobile app that was developed for this pilot study based on our previous research with the target audience of young MSM [18], implementation intention theory [26], and design thinking. The implementation intention theory proposes that the gap between intention and behavior can be effectively bridged by concrete action plans that address when, where, and how the intention can be translated into action [26-28]. Design thinking is a human-centered framework that places the target audience (end user of our mHealth tool) at the center of all key design decisions. Design thinking utilizes empathy to guide a collaborative, codesign process centered on end user needs, wants, and behaviors to create solutions that engage the target audience [29]. The mHealth tool sought to bridge the intention-behavior gap by addressing known barriers to vaccination among young MSM [12,16-18,30]. The tool also served as a bridge between the virtual community and an MSM-affirming health center. Informed by young MSM [18], the tool content included education on HPV, HPV vaccine, prevention of HIV/sexually transmitted infection, and how the health center could help with health insurance enrollment and vaccine cost assistance. Tool functionalities included geolocation/directions to the health center, appointment scheduling, and appointment reminder system.

### Sample and Setting

A convenience sample of young MSM aged 18-26 years was recruited over 6 months (March 2018 to September 2018) via banner and push advertisements primarily posted on 1 mobile MSM dating app that is popular among racially/ethnically diverse men. All advertisements were geolocated to app users in Boston, Massachusetts. Toward the end of the recruitment timeframe, we trialed an additional 1 month of advertisements with another well-known MSM dating app to boost enrollment efforts (our goal was to enroll 200 young MSM). Young MSM who clicked on the study advertisement were directed to the study webpage to assess eligibility and provide informed consent. Participants were eligible if they identified as MSM, were aged 18-26 years, and were a user of our targeted MSM dating app in the Boston area. Eligible and consented individuals were invited to download the study mHealth tool (iOS and Android compatible) to their mobile devices. At the time of download, participants were invited to complete an electronic study questionnaire that collected demographic data and measured knowledge, attitudes, and beliefs related to HPV vaccination. Participants either completed this questionnaire during the initial tool download or returned to completing the questionnaire at the time of their choosing after download (this option was made available to reduce barriers for those for whom time was a constraint).

The partner health center was prepared to receive study participants, enroll them into care, assist with insurance enrollment or vaccine cost assistance programs, and provide HPV vaccine services as medically indicated. HPV vaccination appointments made in the mHealth tool (monitored by the principal investigator) were communicated directly to the health center administrator so that an appointment could be booked as “study participant–HPV vaccination” (as personal identifiers were not collected in the mHealth tool). Once an individual arrived at the center for the scheduled appointment, they identified themselves, and usual care commenced. This health center primarily cares for sexual and gender minority adolescents and adults up to age 29 years, and appointments are largely booked as “same-day” or “walk-in.” Nurses (or other health providers) provided usual vaccine care and education as per the clinic protocol. Participants receiving HPV vaccination at the health center were asked to log their clinic visit on the study-specific clinic iPad. No personal identifiers were collected. Study protocols and procedures were approved by the Fenway Health Institutional Review Board.

### Measures

We sequentially collected the following rates of participation in the study.

1. Accessing the study webpage (collected via Qualtrics)
2. mHealth tool download and usage data (collected within the study tool)
3. Initiation/completion of study questionnaire (collected via Qualtrics)
4. HPV vaccine initiation (collected via study-specific clinic iPad that was linked to Qualtrics or via self-report in the mHealth tool by those who obtained vaccination with another health provider)

The study questionnaire included questions on demographics (eg, race/ethnicity, health insurance status, and employment/student status) as well as previously validated questions on HPV knowledge, attitudes toward HPV and HPV vaccination, subjective norms, perceived behavioral controls, and degree of intention toward vaccination, adapted from our prior work [31-34]. Participants were offered a US $5 gift card plus entry into a prize drawing for a US $75 gift card (1 awarded each month) for completing the questionnaire.

### Data Analysis

The primary outcomes were rates of participation in each sequential study step. Data collected via Qualtrics and through the mHealth tool were managed using Microsoft Excel. Descriptive analyses of study steps (absolute and intermediate completion rates) were completed. Other descriptive analyses included means for continuous variables and percentages for nominal data.

## Results

**Figure 1:**
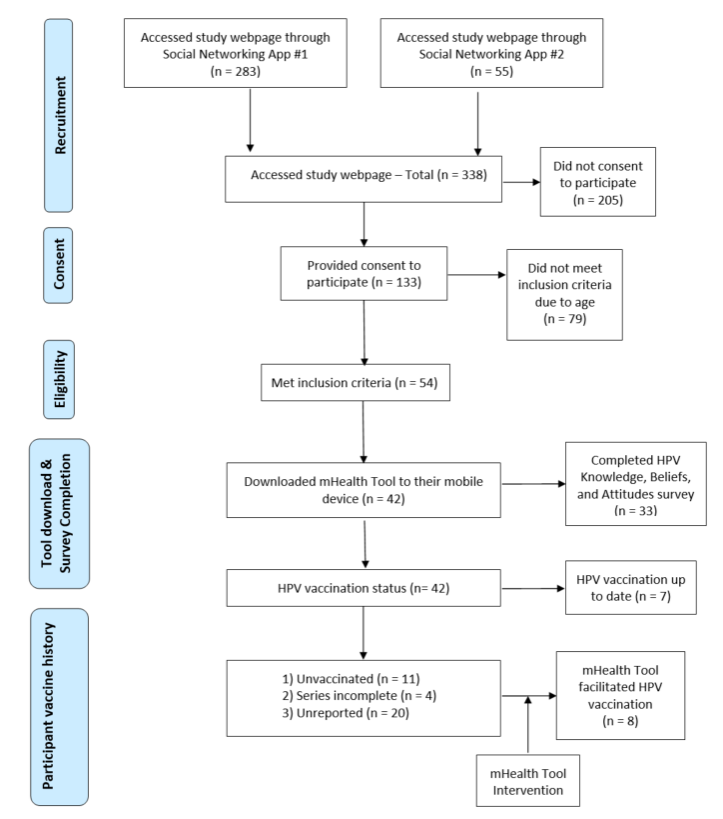
Participant progression though study protocol. HPV: human papillomavirus; mobile health: mHealth.

At the time of recruitment initiation (March 2018), available data from the partnered dating app indicated that approximately 2021 Massachusetts-based users aged 18 to 25 years had logged in during the previous 30 days. During the 6 months of recruitment, a total of 338 potential participants accessed the study web page and engaged in some way. Out of those potential participants, 54 participants met the eligibility requirements and provided informed consent. A total of 42 participants then chose to download the mHealth tool on their mobile devices and participate in the pilot study. Out of these, 33 completed the study questionnaire (Figure 1) and 67% (22/33) completed the questionnaire during the initial mHealth tool download.

The mean age of participants who completed the study questionnaire was 22.7 years. The sample was diverse. For example, 18% (6/33) of the participants reported ethnicity as Hispanic. Race was reported as 9% (3/33) Asian, 6% (2/33) American Indian/Alaska Native, 18% (6/33) Black, 18% (6/33) White, and 18% (6/33) Multiracial; 30% (10/33) participants did not report their race. Out of the 33 participants, 4 (12%) completed some high school, 5 (15%) were high school graduates, 6 (18%) attended some college, 6 (18%) were college graduates, and 36% (12/33) did not report their education level. Health insurance type was reported as 24% (8/33) private, 24% (8/33) public, and 12% (4/33) uninsured; 40% (13/33) participants did not report on their health insurance. Lastly, 30% (10/33) worked full-time; 18% (6/33) were full-time students; 9% (3/33) were part-time students or worked part-time; and 42% (14/33) did not report on their work/student status.

Participants reported variable HPV knowledge (ie, higher knowledge of HPV risk factors and lower knowledge of HPV-related cancer risks for men) and generally positive vaccine beliefs and attitudes (Table 1).

Of the 42 participants who engaged with the mHealth Tool, 7 (17%) were up-to-date with their HPV vaccination, 11 (26%) were unvaccinated, 4 (9%) were not up-to-date on vaccine dosing, and 20 (48%) did not report their vaccination status. Of those who were either unvaccinated, not up-to-date, or did not report status, 23% (8/35) utilized the mHealth tool to facilitate HPV vaccine. Out of these 8 participants, 4 obtained vaccine at the partnered health center, and 4 obtained vaccine at another health center (Figure 1).

Usage data from the mHealth tool revealed that participants primarily accessed (1) the tool’s educational components focused on HPV and the HPV vaccine and (2) the tool’s functionalities that facilitated development of concrete action plans (eg, appointment booking, appointment reminders, and health center geolocation directions).

**Table 1:** Questionnaire data on HPV knowledge, attitudes, and beliefs.^a^

Questionnaire items		Responses, n/N (%)	Responses, n/N (%)
**HPV^b^ knowledge**		Yes	No
	Have you ever heard of the HPV?	24/32 (75)	8/32 (25)
	Has your health care provider ever recommended the HPV vaccine?	19/31 (61)	12/31 (39)
**Attitudes**		True	False
	The use of condoms can help protect you from getting HPV	19/23 (83)	4/23 (17)
	HPV can be spread through oral to genital contact.	19/23 (83)	4/23 (17)
	A person may have HPV and not know it.	21/22 (95)	1/22 (5)
	Both men and women can get HPV.	21/23 (91)	2/23 (9)
	HPV can cause anal cancer.	13/22 (59)	9/22 (41)
	HPV can be cured.	12/22 (54)	10/22 (46)
	HPV can cause cancers of the head and neck.	13/22 (59)	9/22 (41)
	Having a history of multiple sexual partners increases your risk for HPV.	20/22 (91)	2/22 (9)
**Attitudes and Beliefs**		Agree	Disagree/neutral
	Vaccines are important to prevent disease that can be spread person to person.	17/19 (89)	2/19 (11)
	Vaccines should be required for contagious diseases that can be spread person to person.	18/18 (100)	0/18 (0)
	If there was a vaccine that prevented cancer, I would want it.	15/18 (83)	3/18 (17)
	It is safe to get vaccinated for HPV.	14/18 (78)	4/18 (22)
	The HPV vaccine only helps women.	3/18 (17)	15/18 (83)
	It is important for gay men to get the HPV vaccine.	15/18 (83)	3/18 (17)
	My parents would think less of me if I got the HPV vaccine.	4/16 (25)	12/16 (75)
	My friends would think less of me if I got the HPV vaccine.	2/16 (12)	14/16 (88)
	My sexual partners would think less of me if I got the HPV vaccine.	2/16 (12)	14/16 (88)
	It would be hard for me to find time to get vaccinated for HPV.	4/16 (25)	12/16 (75)
	It would be hard for me to get the HPV vaccine because I am afraid of shots.	5/16 (31)	11/16 (69)
	It would be hard for me to find a health center/health provider in order to get vaccinated.	3/16(19)	13/16 (81)
	I am planning on getting the HPV vaccine within the next 30 days	6/16 (38)	10/16 (62)

^a^Findings are reported as n/N (%), where N is the number of people who answered each question, which varied from question to question.

^b^HPV: human papillomavirus.

## Discussion

We aimed to determine if it was possible to recruit young MSM via a dating app for an mHealth (app-based) intervention that would effectively engage young MSM in local health services related to HPV vaccination. The results of this study support the theoretical framework (implementation intention theory), which posits that helping youth create concrete action plans can facilitate health behaviors. Future uses of mHealth tools may be effective in bridging the intention-behavior gap, particularly among adolescents (native technology users).

Of the participants who engaged with the mHealth tool and were either unvaccinated, not up-to-date on vaccine series completion, or did not report their vaccine status, 23% (8/35) utilized the tool to facilitate vaccination with the partnered health center or elsewhere. App usage data documented that participants primarily accessed information about HPV vaccination and used the tools to schedule appointments, geolocate the clinic, and set appointment reminders. Although there are few comparison mHealth studies among young MSM, another web-based educational and email/text message reminder intervention noted success in increasing HPV vaccination rates [35].

Although this study piloted a novel concept for engaging young MSM and for increasing their access to an affirming health center to facilitate HPV vaccination, the study had several limitations. We encountered challenges in study enrollment, and the study was limited by its narrow geographic focus. Enrollment was challenged by participants’ willingness to download the study mHealth tool and engage in the study questionnaire. Thus, we could engage only 42 participants and not 200 as per our original goal. Despite past success engaging young MSM to participate in nonlongitudinal research using dating apps [18], it is possible that many such users may not want to be redirected to an external website or be willing to download a study-specific web-based app where they would be asked to engage over time. Future mHealth research and interventions with young MSM may benefit from adjusted recruitment (eg, recruitment on Facebook or Instagram) and engagement strategies (eg, website-based rather than app-based), which have shown greater evidence of success [35,36] nationally.

Research highlighting varied uses of mHealth tools targeting adolescents and young adults to improve HPV vaccination as well as reduce sexually transmitted infections shows promise [35,37,38]. Our findings suggest that an mHealth tool to support young MSM to create concrete action plans and bridge the intention-behavior gap is possible and may be useful when the health behavior has associated stigmas and when the behavioral goal is singular (eg, HPV vaccination). However, future research is needed to optimize the recruitment and longitudinal engagement strategy of an mHealth tool so that the desired outcomes (eg, increasing HPV vaccination) can be optimally met. Future research should also focus on states or regions with fewer health resources and lower baseline vaccine rates than those in Massachusetts.

The study questionnaire supported previous research [16] that young MSM view HPV vaccination as an important preventative health intervention. However, similar to other studies, results indicate a continued need to enhance knowledge regarding HPV vaccine safety, appropriateness of vaccination for MSM, and HPV-associated cancers in men (eg, anal cancer) [16-18,30]. Interventions aimed at reducing stigmas and increasing access to MSM-affirming health environments that also provide inclusive health education show promise [12,16,18] and are warranted, so that young MSM can comfortably seek health care and gain knowledge of risks and complications associated with HPV disease among MSM.

This pilot study tested feasibility of a theoretically informed mHealth tool designed to reduce health disparities and facilitate HPV vaccination among a sample of young MSM in Boston. We were able to recruit an underserved at-risk population of youth via a mobile online dating platform for an app-based intervention that resulted in in-person health delivery (virtual world to the real world). Considering some of the recruitment challenges, future work is needed to identify optimal strategies to recruit and retain users of mHealth tool technologies to improve the desired outcomes.

## Abbreviations

HPV: human papillomavirus
mHealth: mobile health
MSM: Men who have sex with men
